# Plasma Melatonin Levels, Seasonal Variation, and Sleep Quality: A Stratified Analysis of Adults Aged 25–65 Years Using the Pittsburgh Sleep Quality Index and Actigraphy

**DOI:** 10.3390/ijms27146320

**Published:** 2026-07-16

**Authors:** Irene A. Garcia-Yu, Andrea Crespo-Sedano, César Jiménez-Vaquero, Jesús Gonzalez-Sanchez, Rosario Alonso-Dominguez, Natalia Sanchez-Aguadero, Jose I. Recio-Rodriguez

**Affiliations:** 1 Facultad de Medicina, Universidad de Salamanca, 37007 Salamanca, Spain; igarciayu@usal.es; 2 Instituto de Investigación Biomédica de Salamanca (IBSAL), 37007 Salamanca, Spain; jesusgonzsan@usal.es (J.G.-S.); ralonsod@usal.es (R.A.-D.); ailatan@usal.es (N.S.-A.); donrecio@usal.es (J.I.R.-R.); 3 Complejo Asistencial Universitario de Palencia, 34005 Palencia, Spain; acrespos@saludcastillayleon.es; 4 Escuela Universitaria de Enfermería de Ávila, Universidad de Salamanca, 05003 Ávila, Spain; 5 Facultad de Enfermería y Fisioterapia, Universidad de Salamanca, 37007 Salamanca, Spain

**Keywords:** melatonin, sleep quality, Pittsburgh Sleep Quality Index (PSQI), actigraphy, seasonal variation, adult population

## Abstract

This study aimed to examine plasma melatonin levels, seasonal variation, and their association with sleep quality in adults aged 25–65 years using both the Pittsburgh Sleep Quality Index and actigraphy. A cross-sectional study was conducted in a stratified random sample of 500 adults from Salamanca and Ávila (Spain). Plasma melatonin was quantified by HPLC-DAD-MS/MS, and sleep quality was assessed using the Pittsburgh Sleep Quality Index and a 5-day wrist actigraphy protocol (ActiGraph GT3X+). Participants were stratified by age and sex, and seasonal variation was evaluated. Multivariable analyses were adjusted for relevant confounders. A total of 334 participants with valid melatonin measurements were included (mean age 43 years). Mean plasma melatonin concentration was 14.5 ± 15.9 pg/mL, with higher levels observed in men than women and a decreasing trend with age. A trend toward higher melatonin levels during winter was observed, although the differences were not statistically significant. No significant association was found between plasma melatonin levels and Pittsburgh Sleep Quality Index scores. However, higher melatonin levels were associated with slightly greater sleep efficiency measured by actigraphy. Participants reporting melatonin supplement use showed higher Pittsburgh Sleep Quality Index scores and lower total sleep time compared with non-users. Overall, plasma melatonin levels varied by sex, age, and season but showed no association with subjective sleep quality, while a modest relationship was observed with objective sleep efficiency, suggesting that melatonin alone is not a strong determinant of perceived sleep quality and highlighting the multifactorial nature of sleep regulation in adults.

## 1. Introduction

Melatonin is a hormone primarily produced by the pineal gland. Its synthesis is regulated by the central circadian clock located in the suprachiasmatic nucleus and modulated by environmental light exposure. Under conditions of darkness, melatonin production increases markedly, peaking during the night (usually between 02:00 and 04:00 a.m.), and decreases during daylight hours when light predominates [1,2]. In healthy adults, peak nocturnal plasma levels generally range between 60 and 70 pg/mL, depending on the measurement protocol, while daytime values are significantly lower [1,3]. Evidence also suggests that these rhythms may vary with age, showing a tendency toward reduced nocturnal melatonin secretion as age progresses, although some studies indicate that the basic rhythm persists in healthy older adults [4,5,6]. Furthermore, some investigations have reported sex-related differences, with women exhibiting higher nocturnal melatonin levels or greater rhythm amplitudes; however, findings are not always consistent [7,8].

Sleep quality is a multidimensional construct that can be assessed using both subjective and objective methods. Among subjective measures, the Pittsburgh Sleep Quality Index (PSQI) has been widely validated and is considered a reliable tool for evaluating perceived sleep quality, latency, duration, efficiency, nocturnal awakenings, and daytime dysfunction [9]. However, these evaluations can be influenced by mood, expectations, or individual interpretation of the questionnaire items. To overcome these limitations, objective methods such as actigraphy allow for the continuous monitoring of activity–rest cycles over multiple days under natural conditions, providing information on sleep duration, efficiency, fragmentation, continuity and latency in a non-invasive manner [10,11]. The combination of subjective and objective measures thus offers a comprehensive and robust approach to characterizing sleep quality.

Several studies have examined the relationship between plasma melatonin levels and sleep quality. Some findings in a study including patients with pruritus suggest that reduced or misaligned melatonin concentrations are associated with higher PSQI scores, indicating poorer subjective sleep quality [12]. However, more recent investigations combining PSQI and actigraphy have not consistently reported significant associations, suggesting that the relationship between melatonin and sleep may be modulated by other physiological or behavioral factors [13]. This heterogeneity highlights the need for methodologically rigorous studies with representative samples and appropriate stratification.

Age and sex are particularly relevant factors. With aging, sleep tends to show reduced efficiency, greater fragmentation, and decreased slow-wave sleep, accompanied by an attenuated nocturnal melatonin peak [4,5,6,14]. In addition, sex-related differences have been reported in both the prevalence of sleep disorders and sleep architecture. Women often report poorer subjective sleep quality, and some studies suggest differences in efficiency and latency, observed in both self-reports and objective measures [7,15,16]. These findings emphasize the importance of incorporating age and sex stratification in population-based studies.

Another factor to consider when evaluating plasma melatonin levels is the seasonal effect. This effect is reflected in an increase in the duration of nocturnal melatonin secretion toward the winter solstice (longer nights) and a decrease in its duration toward the summer solstice (shorter nights) [17]. Adamsson et al. [18] observed seasonal variation in melatonin in Sweden (a high-latitude region with large seasonal differences in light), with higher melatonin levels during winter, while daily light exposure occurred mainly in the morning and early afternoon. However, the impact of seasonal changes on melatonin levels at mid-latitudes remains unclear. Nogueira et al. [19] conducted a study involving the general population from mid-latitude regions of the United States and reported that melatonin levels were slightly higher during the winter, although these differences were not statistically significant.

In this context, it is pertinent to systematically analyze the relationship between plasma melatonin levels and sleep quality, considering both subjective and objective dimensions, in a representative adult sample stratified by age and sex. Additionally, analyzing data across different seasons allows for the assessment of seasonal variations in melatonin and their potential impact on sleep patterns. Such a design enables the capture of interindividual variability and the control of potential confounding factors, providing a more accurate understanding of the interaction between physiological processes and the sleep experience.

This study, therefore, aims to analyze plasma melatonin levels in a stratified random sample of adults aged 25 to 65 years, categorized by age and sex, and to evaluate their relationship with sleep quality as measured by the PSQI and actigraphy. Seasonal variations in melatonin will also be considered. This approach is expected to provide solid evidence on the role of melatonin as a biomarker of sleep quality and to explore its potential utility in identifying risk profiles for sleep disturbances within the adult population.

## 2. Results

Of the 500 participants initially included in the study, only 334 had valid melatonin measurements and were therefore included in the present analysis. The resulting sample comprised 334 individuals, with a mean age of 43.7 years. Most participants were either married or in a non-marital union (52.4%), had completed middle or high school, or higher education (89.8%), and were employed (68.3%). The mean BMI was 26.1 ± 5.0 kg/m^2^, and the mean MEDAS score was 8.7 ± 2.2. Only 6.0% of participants reported taking melatonin supplements (Table 1).

### 2.1. Melatonin Plasma Concentration

Overall and age- and sex-stratified plasma melatonin levels are presented in Table 2. The overall mean plasma melatonin concentration was 14.5 ± 15.9 pg/mL, with significant differences between men (17.3 ± 16.9 pg/mL) and women (11.7 ± 14.4 pg/mL). In addition, significant sex-based differences in melatonin levels were observed in the 25- and 65-year-old groups (*p* < 0.05). In the 25-year-old group, men had a melatonin concentration of 15.6 ± 16.0 pg/mL, while women had 8.6 ± 11.3 pg/mL. In the 65-year-old group, men showed a concentration of 12.4 ± 8.2 pg/mL, whereas women had 4.9 ± 6.4 pg/mL.

Furthermore, significant differences in mean plasma melatonin levels were found between age groups (*p* < 0.05), specifically when comparing the 65-year-old group with the 35- and 55-year-old groups, and the 25-year-old group with the 55-year-old group.

No significant differences were observed in mean plasma melatonin levels according to melatonin supplement use, although levels were higher in participants who reported taking melatonin supplements compared to those who did not (18.9 vs. 15.3 pg/mL, respectively). Sensitivity analyses excluding participants who reported melatonin supplement use (*n* = 20) showed consistent results.

### 2.2. Melatonin Levels According to Season

Before comparing plasma melatonin concentrations across seasons, baseline characteristics were examined according to season of assessment. Significant differences were observed for age, sex, marital status, educational level, body mass index, alcohol consumption, and employment status, whereas no significant differences were found for work schedule or melatonin supplement use.

**Figure 1:**
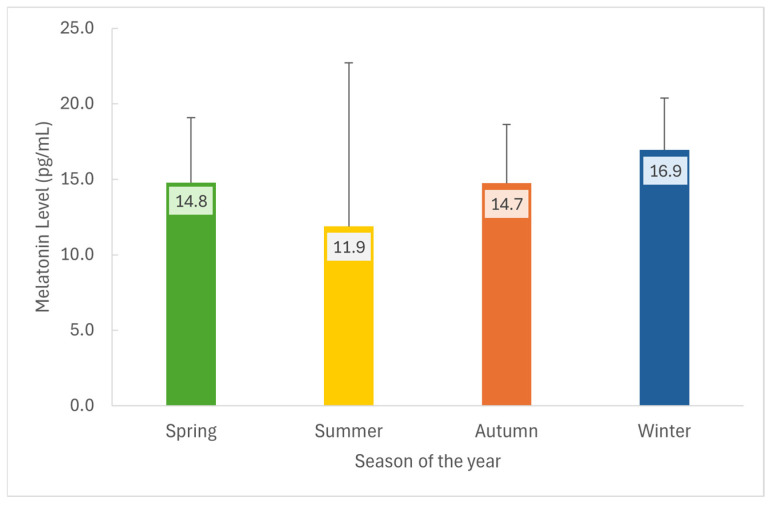
Mean melatonin levels by season. Notes: Adjusted by age, sex, educational level, work schedule, CCI, BMI, LTE score, adherence to the Mediterranean diet score, alcohol consumption, and melatonin supplementation.

Regarding melatonin levels according to the season in which the assessments were conducted, mean plasma melatonin concentrations tended to be higher in winter (16.9 pg/mL; 95% CI: 13.5–20.4) than in summer (11.9 pg/mL; 95% CI: 1.1–22.7). However, the differences observed between seasons were not statistically significant (Figure 1).

### 2.3. Sleep Quality

Sleep quality indicators were analyzed considering the median of plasma melatonin (8 pg/mL) (Table 3). Sleep quality, assessed using the PSQI, showed no significant differences between participants with plasma melatonin levels ≤ 8 pg/mL and those with levels > 8 pg/mL (*p* = 0.896). However, sleep efficiency was significantly higher in the group with plasma melatonin > 8 pg/mL (90.9% vs. 89.8%, *p* = 0.027). A higher WASO was observed in subjects with plasma melatonin levels ≤ 8 pg/mL compared to those with levels > 8 pg/mL (42.2 vs. 38.0 min), although these results were at the threshold of statistical significance (*p* = 0.068). No significant differences were observed in other indicators of sleep quality, including total time in bed, TST, and number of awakenings.

Differences in PSQI scores were observed according to melatonin supplement use. Subjects who reported taking melatonin supplements had higher mean PSQI scores (9.3 points), indicating poorer subjective sleep quality, compared to participants who did not take melatonin supplements (6.4 points) (*p* = 0.006; Cohen’s F = 7.611). In addition, a higher total time in bed was observed in subjects who did not take melatonin supplements (424 min) compared to those who did (353 min) (*p* = 0.001; Cohen’s F = 10.682). Differences were also observed in total sleep time (TST), with higher values in subjects who did not take supplements (381 min) compared to those who did (320 min) (*p* = 0.004; Cohen’s F = 8.246).

## 3. Discussion

This study examined melatonin plasma concentrations and their association with multiple indicators of sleep quality in a sample of adults aged 25–65 years. The overall mean plasma melatonin concentration was 14.5 ± 15.9 pg/mL. However, plasma melatonin concentrations differed by sex, with higher concentrations observed in men (17.3 ± 16.9 pg/mL) compared to women (11.7 ± 14.4 pg/mL). In addition, sex-based differences in melatonin levels were evident in both the 25- and 65-year age groups. Regarding seasonality, a trend toward higher melatonin levels was observed during winter, followed by spring and autumn, in contrast to the lower levels detected in samples collected during summer. While subjective sleep quality, as measured by the PSQI, did not differ according to melatonin levels, individuals with higher plasma levels exhibited slightly greater sleep efficiency as assessed by actigraphy. No significant differences were observed in other sleep quality indicators.

The mean plasma melatonin concentration observed in our study (14.5 ± 15.9 pg/mL), measured between 09:00 and 11:00 a.m., is consistent with the physiological decline in circulating melatonin during the morning hours described in healthy adults [1]. Previous studies have shown that circulating melatonin concentrations vary markedly according to sampling time. Hsing et al. [20] demonstrated higher morning serum/plasma melatonin concentrations in samples collected before 9:00 a.m. compared with those collected after 11:00 a.m., highlighting the importance of sampling time when interpreting circulating melatonin concentrations. Similarly, Nogueira et al. [19], in a cohort of men aged 55–74 years, reported mean serum melatonin concentrations of 7.86 ± 0.70 pg/mL in samples collected between 7:00 and 9:00 a.m., which decreased to 3.92 ± 0.79 pg/mL in samples collected between 10:00 a.m. and 12:00 p.m. Differences in analytical methods may also contribute to the variability in circulating melatonin concentrations reported across studies, as previous epidemiological studies have mainly relied on immunoassays, whereas melatonin concentrations in the present study were quantified using HPLC-DAD-MS/MS. However, comparisons between studies should be interpreted cautiously due to differences in study populations, biological samples, sampling protocols, and analytical methodologies.

Melatonin supplement use has been reported to increase plasma levels of this hormone, even after nocturnal intake, maintaining supraphysiological melatonin levels in the morning [21]. In our study, participants reporting melatonin supplement use showed a similar trend, with numerically higher plasma melatonin concentrations; however, this difference was not statistically significant and should be interpreted cautiously. Previous pharmacokinetic evidence indicates that exogenous melatonin may increase circulating melatonin levels [22]. However, direct comparisons with our findings are limited because relevant characteristics of melatonin exposure, including the timing of supplementation relative to blood collection, dose, and formulation, were not available in this study.

On the other hand, our results show differences in melatonin concentrations between men and women. Recent evidence suggests that sex differences in melatonin physiology may partly reflect differential sensitivity to light and circadian regulation. A controlled laboratory study found that women exhibited greater melatonin suppression in response to moderate evening light exposure than men, and that the timing of dim light melatonin onset (DLMO) varied across menstrual phases, supporting the role of sex hormones in modulating melatonergic responses [23]. Moreover, Vidafar et al. [24] reported enhanced sensitivity of the female circadian system to bright light, implying that environmental light cues may differentially shape melatonin rhythms across sexes. A recent systematic review of sex differences in sleep and circadian disorders further underscores the complexity of neurohormonal sex influences on circadian biology, including potential interactions between reproductive hormones and melatonin secretion [25]. However, in our study, the menstrual phase or menopause in women at the time of sample collection for melatonin measurement, as well as any other sex hormone-related variables, was not recorded.

Participants in the older age group exhibited lower melatonin concentrations compared with the rest of the sample, in line with previous studies and the broader literature, suggesting a gradual, age-related decline in melatonin levels [6,26,27]. As noted by Arendt et al. [1], the decline in age-related melatonin production has been attributed to several factors, including early-life calcification of the pineal gland, impaired noradrenergic innervation to the gland, and reduced light perception due to ocular changes such as cataracts or decreased pupil dilation.

Previous studies have reported seasonal differences in melatonin levels, with higher serum melatonin concentrations observed in winter compared to summer, suggesting that melatonin secretion is influenced by variations in light exposure both throughout the day (circadian rhythm) and across the year [19,28]. Our results show a similar trend, with higher melatonin concentrations in winter, when daylight is reduced, and lower levels in summer, when light exposure is greater. Intermediate values were observed in autumn and spring. However, the proportion of samples collected in summer was notably lower than in the other seasons, which may have limited the ability to detect statistically significant differences in these parameters.

In contrast to our findings, a systematic review and meta-analysis of randomized controlled trials found that exogenous melatonin has beneficial effects on sleep quality as assessed by the PSQI in adults with some specific conditions [29]. However, other studies have not found differences in PSQI following melatonin use, although these were conducted in patients with multiple sclerosis [30,31]. In our study, a shorter TST was observed in participants taking melatonin supplements compared with those who did not, whereas Hsu et al. [31] reported longer TST and a trend towards higher sleep efficiency associated with melatonin use. Nevertheless, only a small proportion of participants in our study (6%) reported taking melatonin supplements; therefore, these findings should be interpreted with caution. Furthermore, melatonin supplement use should not necessarily be interpreted as a marker of a diagnosed sleep disorder. In Spain, melatonin at doses below 2 mg may be marketed as a food supplement, whereas products containing ≥2 mg are classified as medicinal products [32]. Therefore, melatonin supplement use may reflect diverse non-medical reasons rather than the presence of a clinically diagnosed sleep disorder, and the binary classification of supplement use in the present study should be interpreted with caution. In addition, another aspect to consider when evaluating exogenous melatonin intake is its formulation, as this may influence the duration of its effect. As reported by Thanawala et al. [33], sustained-release exogenous melatonin resulted in higher and more prolonged plasma melatonin concentrations compared to immediate-release formulations. However, the type of melatonin supplement used was not recorded in our study.

In addition, it has been suggested that age-related declines in melatonin may contribute to poorer sleep quality [34]. However, our findings did not show significant differences in PSQI scores across melatonin levels. This discrepancy could indicate that, within a physiological range, variations in melatonin alone are not sufficient to meaningfully influence subjective sleep quality. It also supports the idea that sleep quality is multifactorial, with biological, behavioral, and environmental factors likely playing a more prominent role than melatonin alone in determining PSQI outcomes [35].

Melatonin has been associated with certain sleep parameters measured by actigraphy, such as increased sleep duration, in studies involving populations different from ours, including the study by Lubas et al. in survivors of childhood cancer [36]. In contrast, in other populations, such as patients with Alzheimer’s disease, no association was found between melatonin and sleep quality parameters measured by actigraphy [37]. Although differences in sleep efficiency were observed in our study, the magnitude of the effect was modest and should be interpreted with caution.

Our findings may have potential clinical implications. The lack of association between plasma melatonin levels and subjective sleep quality suggests that melatonin alone may not be a reliable biomarker for perceived sleep disturbances in clinical practice. The modest associations observed with actigraphy-derived sleep efficiency indicate that melatonin may have a limited role in certain objective sleep parameters, although its clinical relevance appears small in magnitude. Overall, these results support a cautious interpretation of melatonin levels as an isolated indicator of sleep quality. A more comprehensive evaluation considering multiple biological, behavioral, and environmental factors is likely more appropriate when assessing and managing sleep disturbances.

This study presents several strengths. First, it explores the relationship between melatonin and both subjective (PSQI) and objective sleep-related outcomes, contributing to a more comprehensive assessment of sleep quality. Additionally, the inclusion of actigraphy-derived parameters provides an objective measure of sleep, reducing reliance on self-reported data alone. Another strength is the use of a well-characterized sample, which enhances the internal validity of the findings and allows for a more controlled evaluation of sleep-related variables. Finally, the assessment of circulating melatonin levels provides a biologically relevant biomarker, and urine melatonin has been shown to correlate with serum concentrations [30], supporting the validity of peripheral measures of melatonin.

However, several limitations should be acknowledged. The cross-sectional design does not allow causal inferences regarding the relationship between melatonin and sleep outcomes. In addition, no universally accepted clinical cut-off values exist for circulating melatonin concentrations in adult populations; therefore, plasma levels were categorized according to the sample median, a strategy commonly used in epidemiological studies of hormonal biomarkers. Moreover, melatonin secretion follows a strong circadian rhythm, with peak concentrations occurring during the night, meaning that single daytime plasma measurements may not fully reflect individual nocturnal secretion patterns. In this sense, plasma melatonin represents a single time-point measurement and may not capture the full circadian profile compared with repeated sampling or urinary metabolites. Although alternative approaches, such as salivary or urinary measurements, may provide more integrated assessments of melatonin secretion, plasma determination was selected due to its feasibility and widespread use in clinical and research settings. Furthermore, the relatively modest sample size may have limited statistical power to detect small effects. Moreover, information on melatonin supplement use was limited, as data on dosage, formulation, timing, duration, indication for use, and treatment adherence were not collected; therefore, the potential impact of these factors on circulating melatonin concentrations and the interpretation of supplement-related findings could not be fully assessed. Additionally, the unequal distribution of participants across seasonal groups, particularly the small number of participants assessed during summer, may have reduced statistical power and increased the uncertainty of the seasonal estimates. Therefore, these findings should be interpreted with caution. Sleep quality was partly assessed using self-reported measures, which may be influenced by subjective perception and recall bias. Finally, some potential confounding factors, such as light exposure, chronotype, and certain lifestyle and psychological variables, were not fully assessed, which could have influenced the observed associations.

## 4. Materials and Methods

### 4.1. Design

This is a cross-sectional observational study conducted between May 2022 and April 2024 in the cities of Ávila and Salamanca, which are located in the central region of Spain. The study was registered in Clinical-Trials.gov (NCT05324267), as indicated in the study protocol [38]. The study was conducted in accordance with the Declaration of Helsinki and approved by the Ethics Committee for Drug Research of the Health Areas of Salamanca and Ávila (Spain) (CEIm Code: PI 2021 07 815).

### 4.2. Participants

The study population consisted of 500 adults aged from 25 to 65 years, from the cities of Salamanca and Ávila (Spain).

Participants were selected using stratified random sampling by age and sex, using the health card database of the Health Service of Castilla y León, Spain (SACYL). Individuals were distributed equally between men and women in each of the defined age groups (25, 35, 45, 55, and 65 years), including a total of 100 individuals. Subjects were contacted by telephone, and those who refused to participate were replaced in order to complete the sample size.

**Figure 2:**
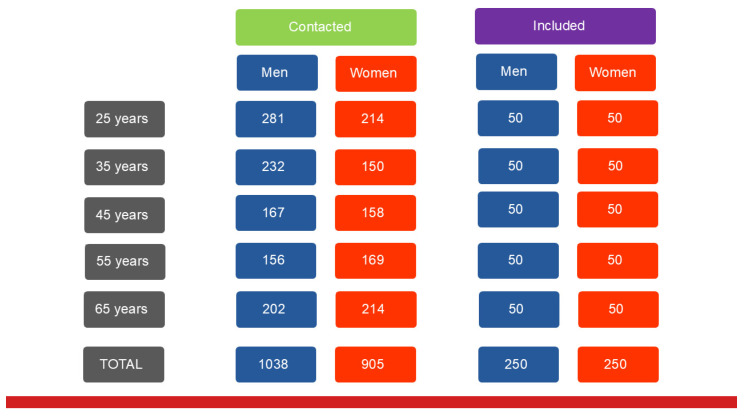
Distribution of participants by age group and sex.

Figure 2 illustrates the participants’ data, stratified by age group and sex. The participants did not take part in the design of the study.

### 4.3. Eligibility Criteria

#### 4.3.1. Inclusion Criteria

Individuals aged 25, 35, 45, 55, or 65 years at the time of sampling, registered in the health card database of the Health Service of Castilla y León, Spain (SACYL), residents of the cities of Salamanca or Ávila (Spain), and those who signed the informed consent to participate in the study and did not meet any of the exclusion criteria, were included.

#### 4.3.2. Exclusion Criteria

Individuals who were terminally ill, those who were unable to attend health centers for the required evaluations, and pregnant women were excluded from the study.

### 4.4. Sample Size Estimation

The sample size was estimated based on expected scores on the PSQI, using data reported by Gómez-García et al. in a study involving Spanish nurses, which found a mean PSQI score of 6.8 points with a standard deviation (SD) of 3.38 [39]. Based on these parameters, a randomized sample of 500 individuals was determined to be sufficient to estimate the population means with a 95% confidence interval (CI) and a precision of ±0.32 points, assuming an approximate SD of 3.38. A 15% loss to follow-up was anticipated and accounted for in the final sample size calculation.

### 4.5. Structure and Planning of Visits

Once the sample was selected, participants were contacted by telephone to offer them the opportunity to participate and to verify inclusion and exclusion criteria. Those who agreed were scheduled for an initial visit with the trained nurse responsible for conducting the evaluations. These visits, held at the Faculty of Nursing at the University of Salamanca and at the University School of Nursing in Ávila (Spain), lasted approximately 60–90 min. During the visit, participants received detailed information about the study, completed interviewer-administered questionnaires, and a signed informed consent form was collected.

The interviewer followed a standardized case report form (CRF) that included sociodemographic, anthropometric, and housing variables, as well as validated instruments such as the PSQI, Generalised Anxiety Disorder-7 (GAD-7), Patient Health Questionnaire-9 (PHQ-9), Mediterranean Diet Adherence Screener (MEDAS), List of Threatening Experiences (LTE), Drug Abuse Screening Test (DAST), Charlson Comorbidity Index (CCI), and alcohol consumption modules. All items were closed-ended or scored using validated scales, ensuring comparability and reproducibility. At the end of the visit, an ActiGraph GT3X+ triaxial accelerometer (ActiGraph LLC, Pensacola, FL, USA) was placed on the non-dominant wrist to be worn for five consecutive days, including at least one weekend day in all cases, and participants were given the self-administered questionnaires. After this period, participants returned to the research center to hand in the accelerometer and completed questionnaires and to undergo a blood draw. The statistical analyst was blinded to the identity of the researcher responsible for conducting the evaluations to reduce bias.

### 4.6. Study Variables

#### 4.6.1. Sociodemographic Variables

Data were collected on age, sex, marital status, educational level, current employment status, and working hours.

#### 4.6.2. Anthropometric Variables

Participants’ weight and height were recorded, and body mass index (BMI) was calculated and expressed in kg/m^2^.

#### 4.6.3. Stressful Events

The LTE [40], a validated 12-item questionnaire assessing exposure to stressful experiences over the past 6 months, including health problems, family conflicts, losses, or work difficulties, was used. Responses were recorded dichotomously (Yes = 1, No = 0). Although no universal cut-off points have been established, higher scores on the LTE indicate greater exposure to stressful events, which are generally associated with increased psychological impact.

#### 4.6.4. Dietary Habits

Adherence to the Mediterranean diet pattern was evaluated using the MEDAS [41], a validated tool consisting of 14 dichotomous (Yes/No) questions. Of these, 12 refer to the frequency of food consumption and 2 to eating habits considered representative of the traditional Spanish Mediterranean diet. Each affirmative response was scored as 1, and each negative response as 0, yielding a total score ranging from 0 to 14.

Melatonin supplement intake was also recorded.

#### 4.6.5. Alcohol Consumption

Data on the type and quantity of alcohol consumed during the past seven days were collected to provide a comprehensive overview of recent consumption patterns. Subsequently, alcohol intake was quantified in grams per week.

#### 4.6.6. Morbidity

The presence of comorbidities was assessed using the CCI [42], a validated tool developed to predict long-term mortality risk in patients with multiple comorbidities. The CCI evaluates 19 chronic conditions, assigning each a score from 1 to 6 based on its associated relative risk. The total score ranges from 0 to 37 and is adjusted by adding one point for each decade of life after the age of 50.

#### 4.6.7. Sleep Assessment

- Sleep Assessment by Actigraphy (ActiGraph GT3X+)

Objective sleep assessment was conducted using the ActiGraph GT3X+ accelerometer (ActiGraph, Pensacola, FL, USA), a validated and reliable device for estimating sleep characteristics in both adult and adolescent populations [43]. Participants wore the device on their wrists for five consecutive days. The system continuously captures accurate and reliable data on sleep/wake cycles, circadian rhythms, and physical activity, generating detailed reports and graphs.

Raw acceleration data were recorded in three axes at a sampling rate of 30 Hz and aggregated into 60 s epochs using ActiLife 6 software (v6.13.15, ActiGraph). Sleep analysis was performed using validated scoring algorithms. The Cole–Kripke algorithm [44] was applied to classify each epoch as sleep or wakefulness, whereas nocturnal sleep periods were identified using the Tudor-Locke algorithm [45], previously validated against polysomnography in adult populations. The Tudor-Locke algorithm defines sleep onset based on the detection of at least five consecutive epochs scored as sleep. Consequently, the estimated bedtime and sleep onset time coincide in automatically detected sleep periods, resulting in a sleep latency value of zero [46]. Therefore, sleep latency was not included among the actigraphy-derived sleep variables analyzed in this study. The following actigraphy-derived sleep parameters were obtained:

Time in bed (TIB): The interval between the time the subject attempts to initiate sleep and the final awakening, including both sleep and wake periods.

Total sleep time (TST): The total amount of time scored as sleep during the time in bed.

Sleep onset time: The clock time at which sleep is first detected within the recording period.

Wake after sleep onset (WASO): The total amount of wake time occurring after sleep onset and before final awakening.

Sleep efficiency: The percentage of TIB spent asleep, calculated as (TST/TIB) × 100.

Number and duration of awakenings: The frequency and cumulative duration of wake episodes during the sleep period after sleep onset.

Sleep fragmentation index: A measure of sleep disruption based on movement or activity counts.

- Sleep Assessment Using the PSQI

The PSQI is a validated and highly reliable instrument specifically designed to assess subjective sleep quality [9]. It consists of 19 self-rated items grouped into seven components: (1) subjective sleep quality, (2) sleep latency, (3) sleep duration, (4) sleep efficiency, (5) sleep disturbances, (6) use of sleeping medication, and (7) daytime dysfunction. Each component is scored on a scale from 0 (no difficulty) to 3 (severe difficulty), with higher scores indicating lower self-reported sleep quality.

The total PSQI score is obtained by summing the seven component scores, yielding a global score ranging from 0 to 21. A higher total score reflects poorer self-reported sleep quality. In the general Spanish population, the PSQI has demonstrated high internal consistency (Cronbach’s α = 0.81), with a sensitivity of 88.63% and a specificity of 74.99% [9,47].

#### 4.6.8. Melatonin Quantification

A blood sample was collected between 9:00 and 11:00 a.m. to determine plasma melatonin concentration. Quantification was carried out by adapting the method described by Viljoen et al. [48]. Plasma melatonin concentrations were determined using high-performance liquid chromatography coupled with diode-array detection and tandem mass spectrometry (HPLC-DAD-MS/MS), following sample preparation by solid-phase extraction (SPE). The analysis was conducted at the Department of Analytical Chemistry, Nutrition, and Bromatology of the University of Salamanca (Spain).

- Sample Preparation

A volume of 1 mL of plasma was diluted with 1 mL of ultrapure water, and 5 µL of indole-3-acetamide (40 ng/mL) was added as an internal standard. The mixture was applied to an SPE cartridge, which was subsequently washed with 2 mL of mobile phase consisting of ultrapure water acidified with 0.1% formic acid (95%) and acetonitrile (5%). The target analytes were eluted with 2 mL of pure acetonitrile. The eluate was evaporated under a stream of air, and the dry residue was reconstituted in 40 µL of mobile phase.

- HPLC-DAD-MS Analysis

The samples were injected into a Waters Acquity UPLC H-Class system coupled to an Xevo TQS micro triple quadrupole mass spectrometer (Waters, Milford, MA, USA) equipped with an electrospray ionization (ESI) source operating in positive mode.

Chromatographic separation was performed on a Luna Omega C18 column (Phenomenex, Torrance, CA, USA; 50 × 2.1 mm, 1.6 µm) using mobile phase A (water containing 0.1% formic acid) and mobile phase B (acetonitrile), at a constant flow rate of 0.7 mL/min. A gradient elution was applied, starting with 95% mobile phase A and 5% mobile phase B. At 4.5 min, the proportion of mobile phase B increased to 20%. At 5 min, the gradient reached 100% mobile phase B, which was maintained until 6.9 min. Subsequently, the initial conditions (95% A and 5% B) were restored at 7 min and maintained until 10 min.

The ionization conditions consisted of an ESI source voltage of +4.0 kV. Multiple reaction monitoring (MRM) transitions were used for quantification. The internal standard (indole-3-acetamide) was quantified using the transition *m*/*z* 175 > 130 (cone voltage: 20 V; collision energy: 13 eV). Melatonin was quantified using the primary transition *m*/*z* 233 > 131 (cone voltage: 30 V; collision energy: 35 eV) and confirmed with the transition *m*/*z* 233 > 174 (cone voltage: 30 V; collision energy: 13 eV).

Quantification was performed using an internal calibration curve specific to each sequence, which included the internal standard. Samples were processed in duplicate, and internal quality controls at various concentrations were applied. The method proved suitable for detecting melatonin at physiological concentrations, demonstrating high precision and sensitivity.

**Figure 3:**
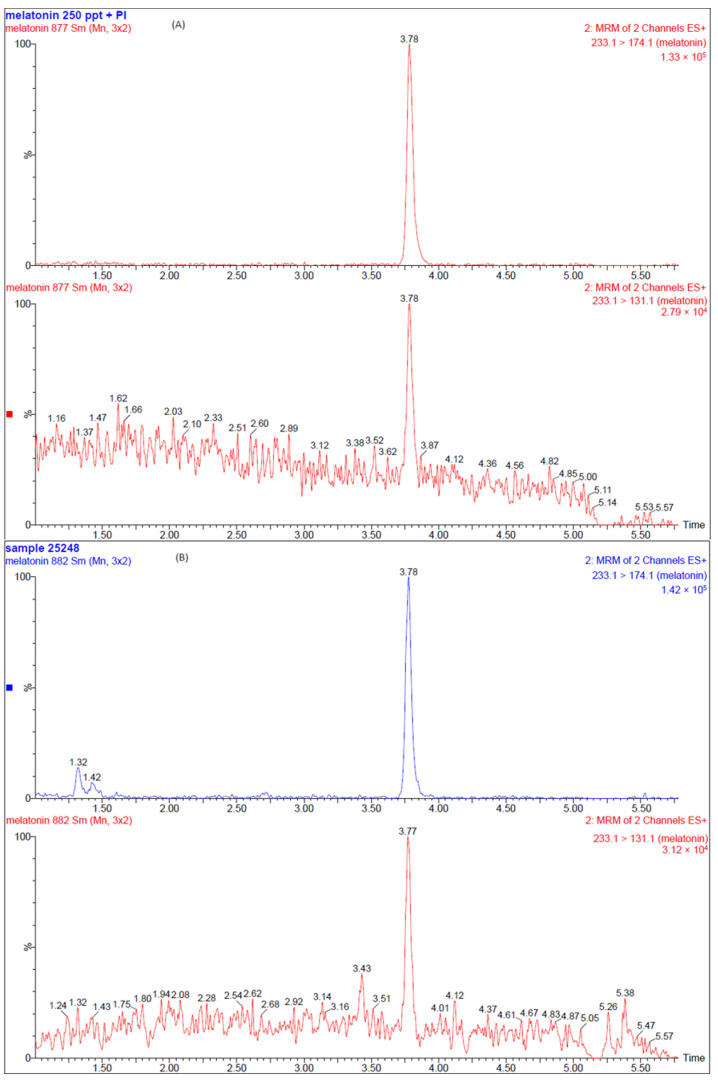
Representative HPLC-DAD-MS/MS chromatograms for plasma melatonin quantification. (**A**) Melatonin standard. (**B**) Representative plasma sample analyzed in the study (sample 25248).

Representative HPLC-DAD-MS/MS chromatograms from a melatonin standard and a representative plasma sample analyzed in the study are shown in Figure 3, providing additional evidence of the specificity of the analytical method used for plasma melatonin quantification.

#### 4.6.9. Other Variables

Regarding seasonal classification, participants were assigned to winter, spring, summer, or autumn according to the exact date of blood collection for plasma melatonin determination. Seasonal groups were defined according to the astronomical seasons in Spain: winter (21 December–20 March), spring (21 March–20 June), summer (21 June–22 September), and autumn (23 September–20 December). In western Spain (including Salamanca and Ávila; approximately 40–41° N latitude), the photoperiod ranges from approximately 9 to 9.5 h of daylight in winter, 11 to 13.5 h in spring, 14.5 to 15.5 h in summer, and 10 to 12 h in autumn.

### 4.7. Statistical Analysis

Statistical analysis was performed according to the study protocol. For this manuscript, the distribution of variables was assessed using the Kolmogorov–Smirnov test. The quantitative variables analyzed showed a normal distribution and are presented as mean ± standard deviation, while categorical variables are expressed as counts and frequency distributions.

For the descriptive analysis of mean plasma melatonin levels by age group and sex, differences according to sex were assessed using Student’s *t*-test, while analysis of variance (ANOVA) was used to evaluate differences among age groups. Post hoc comparisons between age groups were performed using the Bonferroni test.

Finally, as no universally accepted clinical reference values or cut-off points exist for circulating melatonin concentrations in adult populations, the sample was dichotomized into two groups according to the median melatonin concentration (8 pg/mL). Categorizing plasma levels based on the sample median is a commonly used strategy in epidemiological studies of hormonal biomarkers when established thresholds are unavailable. Subsequently, sleep quality indices were evaluated by calculating adjusted estimated marginal means and 95% CI. All analyses were adjusted for potential confounders identified as determinants of plasma melatonin levels or sleep quality indices, including sex, educational level, work schedule, CCI, BMI, LTE score, adherence to the Mediterranean diet (assessed with the MEDAS questionnaire), alcohol consumption, melatonin supplementation, and the season during which the evaluation was conducted. The same analytical approach was applied to compare melatonin levels across seasons. Sensitivity analyses excluding participants who reported melatonin supplement use were also performed. In all cases, a significance level (α) of 0.05 was established as the threshold for statistical significance. Data were analyzed using IBM SPSS Statistics for Windows, version 28.0 (IBM Corp., Armonk, NY, USA).

## 5. Conclusions

In conclusion, this study characterized plasma melatonin concentrations in a sample of adults aged 25–65 years and explored their associations with subjective and objective sleep outcomes. Melatonin levels showed variability according to sex, with higher concentrations observed in men, and a seasonal trend characterized by higher values in winter and lower levels in summer. No association was found between plasma melatonin concentrations and subjective sleep quality as measured by the PSQI. Higher plasma melatonin concentrations were associated with slightly greater sleep efficiency measured by actigraphy, although the magnitude of this association was modest. In addition, participants reporting melatonin supplement use showed higher PSQI scores and lower TST compared with non-users. Overall, these findings suggest that while melatonin may be related to certain objective sleep parameters, its influence on perceived sleep quality appears limited. Sleep outcomes are therefore likely determined by a complex interplay of biological, behavioral, and environmental factors, rather than by melatonin levels alone. Further longitudinal studies with repeated hormonal measurements and comprehensive sleep assessments are warranted to better understand these relationships.

## Figures and Tables

**Table 1:** Clinical and sociodemographic characteristics of the study population.

	Overall (*n* = 334)
Age (years)	43.7 ± 13.9
Female	44.1 ± 14.4
Male	43.4 ± 13.5
Sex, *n* (%)	
Female	168 (50.3%)
Male	166 (49.7%)
Marital status, *n* (%)	
Married/non-marital union	175 (52.4)
Separated/divorced	18 (5.4)
Single	138 (41.3)
Others	3 (0.9)
Educational level, *n* (%)	
Elementary school	34 (10.2)
Middle or high school	132 (39.5)
Higher education	168 (50.3)
Employment status, *n* (%)	
Student	7 (2.1)
Employed	226 (68.3)
Maternity leave	0 (0)
Unemployed	42 (12.6)
Disability to work	5 (1.5)
Retired	47 (14.2)
Home and childcare	4 (1.2)
Missing	3 (0.9)
Work schedule, *n* (%)	
Morning	91 (27.2)
Afternoon	25 (7.5)
Night	3 (0.9)
Shift schedule	32 (9.6)
Part-time schedule (mornings and afternoons)	85 (25.4)
No work	98 (29.3)
CCI	0.34 ± 0.69
BMI (kg/m^2^)	26.1 ± 5.0
LTE score	1.0 ± 1.2
MEDAS score	8.7 ± 2.2
Alcohol (g/week)	49.3 ± 84.7
Melatonin supplementation, *n* (%)	20 (6.0)
Season of the year in which the evaluation was performed, *n* (%)	
Winter	110 (32.9)
Spring	97 (29.0)
Summer	14 (4.2)
Autumn	113 (33.8)

CCI: Charlson Comorbidity Index. BMI: Body Mass Index. LTE: List of Threatening Experiences. MEDAS: Mediterranean Diet Adherence Screener.

**Table 2:** Mean plasma melatonin levels by sex and age group.

	25 Years (*n* = 78)		35 Years (*n* = 84)		45 Years (*n* = 61)		55 Years (*n* = 59)		65 Years (*n* = 52)		Overall (*n* = 334)		
	Men (*n* = 37)	Women (*n* = 41)	Men (*n* = 42)	Women (*n* = 42)	Men (*n* = 34)	Women (*n* = 27)	Men (*n* = 32)	Women (*n* = 27)	Men (*n* = 21)	Women (*n* = 31)	Men (*n* = 166)	Women (*n* = 168)	General (*n* = 334)
Melatonin (pg/mL) *€$&	15.6 ± 16.0	8.6 ± 11.3	19.1 ± 19.4	14.4 ± 16.6	15.9 ± 17.0	13.5 ± 14.7	21.7 ± 18.2	18.2 ± 17.4	12.4 ± 8.2	4.9 ± 6.4	17.3 ±16.9	11.7 ± 14.4	14.5 ± 15.9
	11.9 ± 14.1		16.7 ± 18.1		14.8 ± 15.9		20.1 ± 17.7		7.9 ± 8.1				

Notes: * *p* < 0.05 between men and women. € *p* < 0.05 between men and women aged 25. $ *p* < 0.05 between men and women aged 65. & *p* < 0.05 between age groups (Post hoc analysis contrasts 65 years vs. 35 and 55 years, and 25 years vs. 55 years).

**Table 3:** Sleep quality parameters according to plasma melatonin levels.

	≤8 (Median of the Plasma Melatonin) (*n* = 172, 51.5%)	>8 (Median of the Plasma Melatonin) (*n* = 162, 48.5%)	*p* Value	f Cohen
PSQI score	6.5 (5.9 to 7.2)	6.6 (6.0 to 7.3)	0.896	
Efficiency	89.8 (89.1 to 90.5)	90.9 (90.2 to 91.6)	0.027	4.983
Total minutes in bed	417 (404 to 431)	423 (411 to 436)	0.530	
TST	375 (362 to 388)	381 (369 to 394)	0.513	
WASO	42.2 (39.0 to 45.3)	38.0 (34.9 to 41.0)	0.068	3.355
Number of awakenings	15.1 (14.2 to 16.1)	14.3 (13.3 to 15.2)	0.234	
Average awakening length	3.0 (2.8 to 3.2)	2.8 (2.5 to 3.0)	0.229	
Sleep fragmentation index	24.3 (23.1 to 25.6)	24.2 (23.0 to 25.5)	0.925	
Movement index	13.4 (12.8 to 14.0)	13.0 (12.5 to 13.6)	0.470	
Fragmentation index	10.9 (10.0 to 11.7)	11.1 (10.2 to 11.9)	0.699	

Notes: PSQI: Pittsburgh Sleep Quality Index score; WASO: wake after sleep onset; TST: total sleep time. Adjusted by age, sex, educational level, work schedule, Charlson comorbidity index, body mass index, LTE score, adherence to the Mediterranean diet score, alcohol consumption, melatonin supplementation, and season of the year in which the evaluation was performed.

## Data Availability

The original contributions presented in this study are included in the article. Further inquiries can be directed to the corresponding author.

## Abbreviations

The following abbreviations are used in this manuscript:

PSQI	Pittsburgh Sleep Quality Index
SACYL	Health Service of Castilla y León
SD	Standard deviation
CI	Confidence interval
CRF	Case report form
GAD-7	Generalised Anxiety Disorder-7
PHQ-9	Patient Health Questionnaire-9
MEDAS	Mediterranean Diet Adherence Screener
LTE	List of Threatening Experiences
DAST	Drug Abuse Screening Test
CCI	Charlson Comorbidity Index
BMI	Body mass index
TIB	Time in bed
TST	Total sleep time
WASO	Wake after sleep onset
HPLC-DAD-MS/MS	High-performance liquid chromatography coupled with diode-array detection and tandem mass spectrometry
SPE	Solid-phase extraction
ESI	Electrospray ionization
MRM	Multiple reaction monitoring
ANOVA	Analysis of variance
